# Protein Kinase A Signaling in Cortisol Production and Adrenal Cushing’s Syndrome

**DOI:** 10.3390/cells15010063

**Published:** 2025-12-29

**Authors:** Abhishek Kumar, Abhimanyu Sharma, Mitchell H. Omar

**Affiliations:** Department of Biochemistry, Molecular Biology, and Biotechnology, University of Nevada, Reno, NV 89557, USA

**Keywords:** cyclic AMP, cAMP, PKA, protein kinase A, adrenal, cortisol, glucocorticoids, Cushing

## Abstract

**Highlights:**

**What are the main findings?**

- Disruptions to cAMP/PKA signaling in adrenal cells can cause chronically elevated cortisol levels, known as Cushing’s syndrome.
- Synthesis of the mutations and their impacts suggests that elevated and/or ectopic PKA activity drives pathology, including augmented cell proliferation and overactive cholesterol processing.

**What are the implications of the main findings?**

- Elucidation of the specific roles for type I versus type II PKA, as well as which subcellular populations of the kinase control cortisol production and proliferation, will lead to improved understanding of pathology.

**Abstract:**

The adenosine 3′,5′-cyclic monophosphate–protein kinase A (cAMP-PKA) signaling pathway is highly utilized in human physiology. It is a crucial component of development and is vital to cellular function in nearly all tissues. Indeed, genetic mutations to cAMP-PKA machinery are found in many pathologies, including multiple cancers, cardiac myxoma, neurodevelopmental disorders, and hypercortisolism. Cyclic AMP and PKA were first identified as vital components in cortisol synthesis over 50 years ago, yet the cellular mechanisms connecting PKA to cortisol production are still not well understood. This article will review evidence for PKA’s roles in adrenal gland zona fasciculata steroidogenesis and consider recent studies of the stress hormone disease adrenal Cushing’s syndrome to synthesize a current model for cAMP-PKA actions in cortisol production.

## 1. Cortisol in Health and Disease

**Figure 1:**
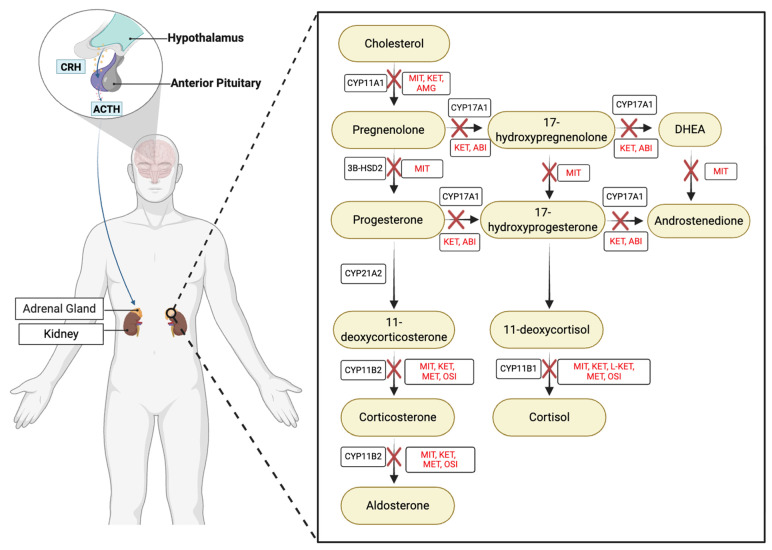
Overview of the hypothalamic–pituitary–adrenal axis and adrenal steroidogenesis pathway. Corticotropin-releasing hormone (CRH) from the hypothalamus stimulates pituitary ACTH release, which activates cortisol synthesis in the adrenal cortex. The zoomed-in view illustrates key steroidogenic steps from cholesterol to cortisol and highlights the inhibitory sites of drugs targeting this pathway. Abbreviations: CYP11A1, cytochrome P450 family 11 subfamily A member 1; CYP17A1, cytochrome P450 family 17 subfamily A member 1; CYP21A2, cytochrome P450 family 21 subfamily A member 2; CYP11B1, cytochrome P450 family 11 subfamily B member 1; CYP11B2, cytochrome P450 family 11 subfamily B member 2; 3β-HSD2, 3β-hydroxysteroid dehydrogenase; DHEA, dehydroepiandrosterone; MIT, mitotane; KET, ketoconazole; L-KET, levoketoconazole; MET, metyrapone; OSI, osilodrostat; AMG, aminoglutethimide; ABI, abiraterone.

The human stress response results in activation of the hypothalamic–pituitary–adrenal (HPA) axis and acute production of the adrenal hormone cortisol (Figure 1). This steroid circulates throughout the body to modify numerous organ and cellular functions, such as downregulation of the digestive system, suppression of the immune system, increased glycogen breakdown, and downregulation of glucose uptake by adipose tissue [1]. These changes result in increased blood sugar for ready availability to the brain, the muscles, and the circulatory system, all needed to respond to and repair from fight or flight situations [1]. Cortisol release also contributes to the circadian rhythm of mammals, controlling arousal and cycling between low and moderate circulating levels in a stereotyped pattern throughout the day [2]. Thus, cortisol production is a vital component of human physiology. However, dysregulation of this glucocorticoid is pathological. Chronically elevated levels of cortisol are associated with increased risk of cardiovascular disease, cancer, and dementia, and can cause pathologies such as depression and Cushing’s syndrome, a disease of chronic cortisol overproduction [3,4,5].

Endogenous Cushing’s syndrome (cases not caused by glucocorticoid medications) can be subdivided into two main categories. Adrenocorticotropic hormone (ACTH)-dependent cases arise from overproduction of the cortisol-stimulating peptide ACTH, most often by either the pituitary gland or ectopic tumors. ACTH-independent, or adrenal, Cushing’s syndrome results from hyperplasia or tumors of zona fasciculata cells of the adrenal cortex [6]. Early signs of Cushing syndrome can often be nonspecific, such as fatigue, mild weight gain, and mood changes [7]. As cortisol excess continues, the classic physical features become more apparent, including central obesity, facial rounding (‘moon face’), and a dorsocervical fat pad (‘buffalo hump’) [8]. Skin changes such as purple striae, thinning of the skin, and easy bruising occur due to cortisol’s catabolic effects on collagen [9]. Women may experience hirsutism, menstrual irregularities, or infertility, while men often develop reduced libido and erectile dysfunction [10]. Additional features include muscle weakness, osteopenia or osteoporosis, hypertension, glucose intolerance, and psychiatric disturbances [11,12]. Overall, individuals with Cushing’s syndrome have a 3.5–5× higher risk of death versus those with normal cortisol levels [13].

The progression of Cushing’s syndrome is typically gradual, with early symptoms often mistaken for common lifestyle-related issues [12]. As elevated cortisol exposure persists, metabolic disturbances worsen, insulin resistance may progress to type 2 diabetes, and persistent catabolism leads to muscle loss and osteoporosis [12]. Over time, patients develop severe cardiovascular complications, including hypertension, dyslipidemia, and increased risk of thromboembolic events [11]. Long-term hypercortisolism also exerts immunosuppressive effects, predisposing individuals to frequent infections [9]. Neuropsychiatric symptoms may intensify, leading to cognitive impairment and depression, further diminishing quality of life [7]. If untreated, Cushing’s syndrome can result in severe morbidity and premature mortality due to metabolic and cardiovascular complications or suicide [9,12].

Early recognition of hypercortisolism is crucial to prevent irreversible organ damage. Diagnostic evaluation typically involves biochemical tests such as the dexamethasone suppression test, late-night salivary cortisol, and 24-h urinary free cortisol measurement [10]. Once confirmed, management aims to reduce cortisol production or block its effects, either through surgical removal of the underlying tumor or medicinal therapies [14,15]. Successful treatment generally reverses many features of Cushing’s syndrome; however, some long-term effects such as bone loss, metabolic dysfunction, and cardiovascular risk may persist [12]. Therefore, early intervention markedly improves outcomes and lowers mortality [7,9].

Pharmacologic therapy plays a crucial role in patients who are not surgical candidates or as an adjunct to surgery (Figure 1). Unfortunately, a common drawback of the current steroidogenesis drugs is the failure to inhibit cholesterol import into mitochondria. In the event of elevated ACTH or augmenting alterations in upstream adrenal cell signaling pathways, inhibition of downstream steroidogenesis enzymes without suppressing cholesterol import causes overproduction of steroid intermediates such as 11-deoxycorticosterone (11-DOC) and drives hypertension [16]. Moreover, the available treatments do not improve symptoms for all patients and can cause debilitating side effects and decreased quality of life [17]. A deeper understanding of the cellular signaling mechanisms that mediate upstream cortisol synthesis signaling is needed to develop amenable strategies for gaining control of cholesterol import into mitochondria.

**Figure 2:**
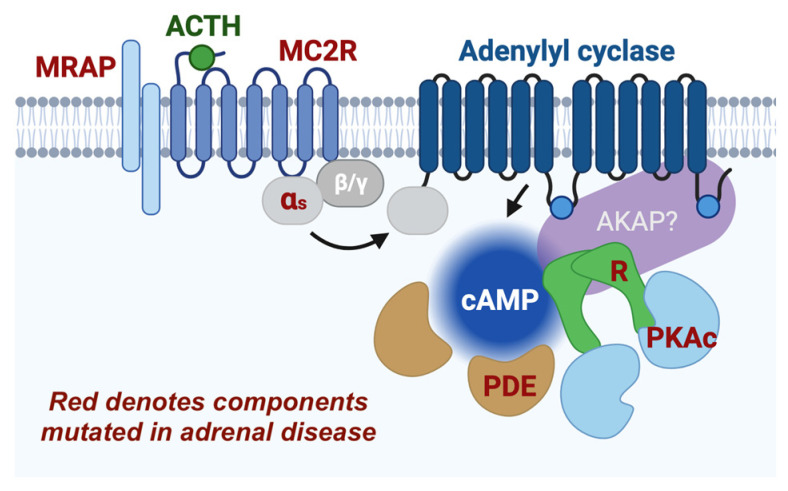
ACTH-stimulated mobilization of the cAMP-PKA pathway in adrenal zona fasciculata. Red text signifies components mutated in adrenal stress disorders. MRAP, melanocortin 2 receptor accessory protein; R, PKA regulatory subunit; PDE, phosphodiesterase. The AKAP (A-kinase anchor protein) is hypothetical. Made with BioRender 2025.

Mechanistically, we know that cortisol is produced as a result of HPA axis activation (Figure 1). In the latter part of this hormonal response to stress, the anterior pituitary releases ACTH into the blood. This hormone binds the adrenal melanocortin 2 receptor (MC2R), a Gαs G protein-coupled receptor (GPCR), and initiates production of cAMP via activation of adenylyl cyclase. The resulting focal cAMP signal activates local populations of PKA, which is a central and required component of cortisol production (Figure 2) [18,19]. While the actions of ACTH-activated PKA have been investigated for over 50 years, our understanding of this kinase in the adrenal gland is still lacking in terms of molecular mechanisms. The present review summarizes studies of cAMP and PKA signaling in cortisol-producing zona fasciculata cells in the adrenal gland as well as the closely related MA10 Leydig cells that produce the steroid testosterone.

## 2. Mechanisms of Protein Kinase A Signaling

PKA is a highly conserved and nearly ubiquitous serine/threonine kinase. It is activated by cAMP and inhibited by the endogenous heat-stable cAMP-dependent protein kinase inhibitor (PKI) [20,21,22]. PKA is a versatile and widely utilized enzyme; it phosphorylates hundreds of basophilic substrates and is expressed in almost every cell in your body [23]. Over sixty years of research on PKA has revealed its roles in numerous cellular and organismal physiologies, including metabolism, learning and memory, cardiac function, and response to a diverse array of hormones [24]. As a reflection of this importance, disruptions to PKA signaling underlie pathologies such as neurodevelopmental disorders, cancers, cardiac myxoma, and adrenal dysfunction [25,26,27]. This kinase is versatile on a subcellular level, as well. Cells use various A kinase anchoring proteins (AKAPs) to compartmentalize PKA to discrete subcellular locations with particular substrates and signaling enzymes. In this way, a cell’s expressed PKA is subdivided into several subpopulations that each govern a distinct cellular process [28].

Structurally, PKA is a dimer of dimers, with two regulatory subunits bound to one another as well as one catalytic subunit each [29]. In humans, there are five genes that can encode for the catalytic subunit (*PRKACA*, *PRKACB*, *PRKACG*, *PRKX*, and *PRKY*), four different regulatory subunits (*PRKAR1A*, *PRKAR1B*, *PRKAR2A*, and *PRKAR2B*), and over 60 identified genes for AKAPs [28,30]. The alpha and beta isoforms are the most prevalent of the catalytic subunits (PKAc) [30]. At rest, PKAc is bound tightly to its regulatory subunit (R) in an inhibitory interaction. Kinase activation occurs when cAMP levels increase in immediate proximity to PKA. The second messenger binds to regulatory subunits, which each contain two cAMP-binding domains. This event weakens the inhibitory interaction between regulatory and catalytic subunits, allowing the catalytic subunit to bind and phosphorylate nearby substrates and thus drive cellular processes [31,32]. In contexts such as the adrenal gland during acute stress, concentrations of cAMP may be strong enough to prolong the weakened interaction between PKAc and R subunits, thereby enabling more distal phosphorylation events. However, this has not been demonstrated in physiological conditions. Alternative manners of PKA regulation include cAMP-independent mechanisms such as the activating interaction between R subunits and the calcium-binding protein S100A1, and the inhibitory interaction between PKAc and the GPCR smoothened [33,34].

The isoform of the R subunit determines whether PKA is type I (RIα and RIβ) or type II (RIIα and RIIβ). While there are several documented functional differences between alpha and beta isoforms, the major differentiating factors exist between the two main types [29]. Compared to type II R subunits, type I R subunits have a higher affinity for cAMP, leading to a lower threshold of activation in in vitro studies [35,36,37,38]. RI also cannot be autophosphorylated on its inhibitory sequence, whereas RII subunits contain a serine that is phosphorylated by the bound catalytic subunit [29]. Furthermore, it is the R subunit that is directly recruited to AKAPs, with each AKAP in this large family displaying a different degree of preference for type I or type II regulatory subunits [28,39,40]. Since each AKAP associates with distinct combinations of cellular machinery at discrete subcellular locations, type I and type II PKA are spatially segregated and assigned to different cellular processes.

## 3. PKA Is a Vital Component of Cortisol Production

Since the late 1950s and 60s, cortisol production by the adrenal gland has been known to require cAMP as a messenger connecting ACTH to steroidogenesis [41,42,43,44]. Elegant studies and deductions from several pioneering labs elucidated that ACTH binds extracellularly to its receptor (now known as the melanocortin 2 receptor, MC2R), which leads to activation of adenylyl cyclase [19,45]. Early efforts also used puromycin and cycloheximide injections in rodents to determine that protein synthesis is required for this hormone-stimulated event [46]. In terms of kinase involvement, these studies preceded much of early kinase research. Glycogen phosphorylase activation was indicated early on and tied to cAMP synthesis through experiments conducted in bovine adrenal slices [41]. Evidence that PKA is the major target of the cAMP signal began accumulating soon after discovery of the kinase by Krebs and Fischer [47]. Garren, Gill, and Walton investigated how cAMP binds to different subcellular fractions of adrenal tissue. The authors found that while the second messenger had broad binding to most fractions, the highest associations were with cytosol and smooth endoplasmic reticulum [48]. These studies were not only foundational for understanding adrenal production of glucocorticoids, but for understanding PKA activation in general. They went on to demonstrate that cAMP bound a “receptor” protein (now known to be regulatory subunits), and then used sedimentation assays to find that presence of the cyclic nucleotide-controlled dissociation of this receptor from a protein with kinase activity. Importantly, this study found that cAMP-dependent dissociation resulted in activation of the kinase [48,49]. In retrospect, this study laid the foundational work for understanding much about PKA action in cortisol synthesis and provided early evidence that this kinase may have roles broader than lying upstream of glycogen phosphorylase. Indeed, many studies since have investigated the substrate targets of PKA in response to ACTH [50,51,52,53].

Another important contribution to cementing PKA’s roles in steroidogenesis was analysis of Y1 adrenal cells [54]. This line was made from a mouse adrenocortical tumor and enabled many mechanistic studies not readily feasible in acute tissue samples. Schimmer and colleagues in particular utilized isolated Y1 mutants to define components of the ACTH-stimulated pathway via a combined biochemical and genetic approach [19]. This work established and corroborated functional contributions for many mediators of ACTH actions including MC2R, adenylyl cyclase, and PKA, and provided evidence for PKA-controlled gene expression in cortisol synthesis [55,56].

## 4. PKA and StAR

One of the key mechanisms of PKA’s critical role in hormone production is its regulation of the mitochondrial cholesterol importer steroidogenic acute regulatory protein (StAR). StAR was first identified in the early 1980s by Orme-Johnson and colleagues as an adrenal protein that is produced in response to ACTH or cAMP stimulation [57]. This group went on to show that StAR is mitochondrially associated and phosphorylated in stimulated cells [58,59]. By performing experiments in the presence of chloramphenicol as a mitochondrial protein synthesis inhibitor, they were able to infer that StAR is synthesized in the cytosol and transported to mitochondria [59]. This group also established that StAR is induced concurrently with steroid synthesis in other steroidogenic tissues, including Leydig cells of the testes [60].

Soon after this, MA-10 Leydig cells were used extensively by Stocco and colleagues to uncover further details of StAR and its impacts on steroidogenesis. Their early efforts solidified that the MA-10 mitochondrial protein induced during steroid production is the same protein identified in ACTH-stimulated cortisol production by the adrenal glands [61,62,63]. Upon isolating and cloning this protein from MA-10 cells, they conducted the important experiment of overexpressing StAR in the absence of stimulation. Their results indicated that this protein is sufficient to promote steroidogenesis and thus named it StAR to reflect its function [64]. Related studies established that PKA can phosphorylate StAR at serine 195 and that this event increases the efficiency of the transporter [53]. This set of experiments expressed a S195A mutant version of StAR in COS-1 cells and demonstrated that lack of phosphorylation at this site decreases steroid production by ~50%. A similar study was later performed in StAR knockout mice wherein replacement with a wild-type StAR fully rescued steroid production but use of an alanine mutant failed to rescue a ~40% decrease in testosterone and an over 50% deficit in corticosterone production [65]. Presumably, this in vivo event is mediated by PKA as well.

How PKA might be directed to StAR was addressed a decade later. Based on StAR’s subcellular localization, it was hypothesized that the recently discovered mitochondrial AKAP1 may organize the hormone-stimulated phosphorylation of StAR [66]. Loss of AKAP1 via siRNA knockdown led to over 50% reduction in StAR protein levels without affecting *StAR* mRNA. Later work by this group found that the K-Homology (KH) motif of AKAP1 recruits *StAR* transcripts to the mitochondria, suggesting a requirement for local translation [67]. Loss of AKAP1 also greatly decreased mitochondrial RIIα protein abundance without visibly changing its cytosolic levels or RI levels at either location [66]. Additional investigation is needed to decouple the mRNA-binding and PKA-recruiting functions of AKAP1 in assessments of StAR phosphorylation. Related studies by this group produced evidence for two different forms of PKA-mediated StAR regulation. The experiments demonstrated induction of CREB phosphorylation and StAR protein levels by RI-activating cAMP analogs, whereas StAR phosphorylation was promoted by RII-activating analogs [68]. It is, however, important to note that cAMP analog selectivity is imperfect and should be considered when interpreting experimental results [69,70,71].

ACTH-dependent transcriptional regulation of steroidogenic genes is reviewed in [18]. Based on the above data, we can infer that transcription factors (TFs) downstream of PKA are activated by type I complexes of the kinase. In the future, it will be important to elucidate the specific signaling interactions, protein translocations, sites of phosphorylation events, and AKAP(s) responsible for coordinating PKA-mediated TF regulation. The above data also suggest that rapid induction of cortisol synthesis in the acute response to stress likely occurs through type II PKA phosphorylation of StAR, although type I roles in this event have not been systematically ruled out. Evidence for ERK1/2 involvement in this process has also been shown [72]. How a plasma membrane-localized MC2R receptor rapidly leads to mitochondrial protein phosphorylation is an intriguing question. It is possible that mitochondria involved in cholesterol import are already associated near the plasma membrane in steroidogenic cells and therefore responsive to ACTH-stimulated cAMP pools at the periphery. However, demonstrations of GPCR internalization and subsequent vesicle-associated pools of cAMP have opened further mechanistic possibilities regarding adrenal PKA activation [73,74]. Answering these spatiotemporal questions will help define the relationship between PKA and StAR and their indispensable roles governing cortisol synthesis.

## 5. cAMP Signaling in Adrenal Disease

Recently, deeper insights into the cell signaling mechanisms that control adrenal glucocorticoids have come from studies of disease. Numerous cortisol disorders exist, spanning from loss of cortisol production in adrenal insufficiency to steroid overproduction in multiple adrenal diseases that cause Cushing’s syndrome. In the case of adrenal insufficiency, disrupting mutations in MC2R or the melanocortin 2 receptor accessory protein (MRAP) account for many cases [75]. In diseases with overproduction of cortisol, the list is more diverse; however, over half of adrenal Cushing’s patients carry genetic mutations associated with the cAMP-PKA pathway (Figure 2) [75,76]. In the sections below, we review major genetic causes of hypercortisolism, including disruptions to receptor complexes, alterations to cAMP degradation machinery, and PKA subunit variants.

## 6. Receptor Complexes

Upstream signaling errors that cause Cushing’s syndrome include ectopic expression of GPCRs such as gastric inhibitory polypeptide (GIP) receptor, beta-adrenergic receptors, or the luteinizing hormone receptor [77]. Ectopic expression of one of these receptors in the zona fasciculata renders cells aberrantly responsive to the wrong hormone and thereby produces cortisol when the body is signaling for a response from different tissues. For instance, GIP is released in response to food ingestion. Cushing’s patients expressing the GIP receptor in cortisol-producing cells resultingly experience a spike in stress hormone levels each time they eat [77]. Furthermore, these hormones are not negatively regulated by increased circulating levels of cortisol, as is the case for ACTH, and therefore glucocorticoid production persists without a coupled off switch.

Immediately downstream of GPCRs are the G-proteins themselves. G-protein mutations driving adrenal disease were identified in McCune–Albright syndrome patients, whose symptoms include hypercortisolism [78]. This study identified activating mutations in the adenylyl cyclase-stimulating G-protein Gαs. Mutations in the gene for Gαs, *GNAS*, account for 7% of McCune–Albright patients with Cushing’s syndrome and underlie ~17% of adrenal Cushing’s syndrome cases [79,80]. The identified protein variants, including R201C/R201H and Q227L versions of Gαs, are found to inhibit the enzyme’s intrinsic GTPase activity. These mutations lock the subunit in its active GTP-bound conformation, thereby driving persistent adenylyl cyclase activation and increased cAMP generation [81].

Gαs contains three dynamic switch regions (Switch I, II, and III) that undergo conformational rearrangement upon nucleotide exchange. In the GDP-bound inactive state, the R201 residue donates the η1 and η2 ammonium groups to form a salt bridge with E50 in the phosphate-binding loop, which in turn prevents E50 from contacting R258 and R265 in Switch III. Disruption of these Switch I–Switch III interactions inhibit formation of the extended H-bond network required for Gαs activation [82]. R201C is the most frequent cancer-causing mutation across all heterotrimeric G proteins [82]. Replacement of arginine with cysteine removes the η1 and η2 ammonium moieties, freeing E50 to interact with R258 and R265 in Switch III and mimicking the structure of the GTP-bound active state. The E50–R258–R265 interaction links Switch III to Switch II residues, promoting formation of the H-bond network that stabilizes Gαs in an active-like conformation [82]. Furthermore, Gαs-R201C variants have reduced affinity for Gβγ. This allows Gαs-R201C subunits to bind and activate adenylyl cyclase in the presence of Gβγ subunits, which is not observed with wild-type Gαs [82].

## 7. Phosphodiesterases

**Figure 3:**
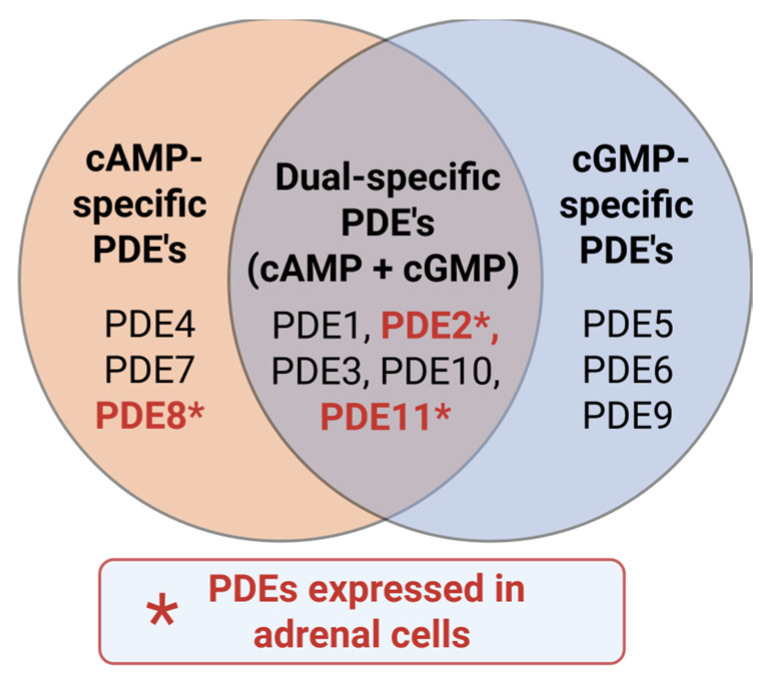
Classification of PDEs based on substrate specificity.

Activation of adenylyl cyclase leads to local production of diffusible cAMP, using ATP as a substrate. Cells utilize multiple phosphodiesterases (PDEs) to convert cAMP and the structurally related secondary messenger cGMP back into non-cyclic nucleotides, therein turning off hormone-induced stimulation of cyclic nucleotide-dependent pathways [83,84]. Thus, the intracellular concentration of cAMP and PKA activity are determined by the balance between adenylyl cyclase and phosphodiesterase activities [85]. A vital concept regarding PDEs in cAMP-dependent actions of PKA is their role in shaping pools of acutely synthesized cyclic nucleotide [86]. Upon stimulation by adenylyl cyclase, PDEs immediately limit the range of the second messenger into discrete nanodomains within the cell. This synergizes with AKAPs to focus PKA action and thereby avoid signaling pathway crosstalk [28]. The PDE superfamily comprises 11 gene families (*PDE1*–*PDE11*) that are structurally related but functionally distinct, with multiple isoforms that selectively hydrolyze cAMP, cGMP, or both (Figure 3) [87]. Among these, *PDE8B* and *PDE11A* are highly expressed in the adrenal cortex [88,89]. *PDE2A* is predominantly expressed in zona glomerulosa tissue of the adrenal gland and its protein product contributes to cAMP–cGMP cross-talk through cGMP-mediated degradation of cAMP, integrating multiple hormonal inputs [90]. Several lesions in these adrenal PDE genes have been identified in cortisol-producing tumors and may be important for understanding pathology in Cushing’s syndrome patients [89,91,92].

### 7.1. PDE2A

**Figure 4:**
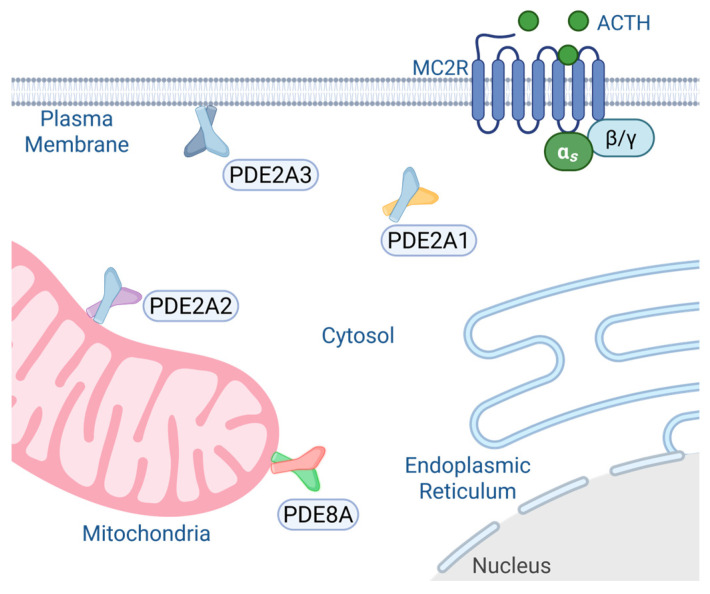
Subcellular localizations of PDEs in adrenal cells. Made with BioRender 2025.

PDE2A, a dual-specific phosphodiesterase, has three main isoforms (PDE2A1–3). Differences in their amino-terminal regions lead to distinct subcellular localizations of each isoform [93]. PDE2A1 is localized in the cytosol, PDE2A2 is found in the mitochondria where it interacts with the mitochondrial contact site and cristae organizing system (MICOS) complex, and PDE2A3 is localized at the plasma membrane (Figure 4). PDE2A1 functions as a homodimer composed of three distinct structural regions: an N-terminal regulatory domain, a central catalytic core, and a C-terminal tail. The N-terminal domain contains two GAF motifs, GAF-A and GAF-B, with GAF-A mediating dimerization and GAF-B harboring the cGMP-binding site essential for allosteric activation. The catalytic domain hydrolyzes both cAMP and cGMP. PDE2A is highly expressed in the zona glomerulosa cells of the adrenal gland [93]. Under conditions of increased blood pressure, cardiac myocytes release atrial natriuretic peptide (ANP), which stimulates the zona glomerulosa cells of the adrenal gland to suppress the release of aldosterone, a hormone that regulates blood pressure [94]. Upon atrial natriuretic peptide (ANP) binding to its receptor, guanylyl cyclase is activated, leading to elevated intracellular cGMP levels. cGMP subsequently binds to the GAF-B domain of PDE2A1 and enhances its cAMP hydrolytic activity. This results in reduced cytosolic cAMP concentrations, attenuation of protein kinase A (PKA) signaling, and ultimately suppression of aldosterone synthesis [95].

In hypercortisolism patients, *PDE2A* mRNA and PDE2A protein levels have been found to be upregulated in adrenocortical tumors harboring *CTNNB1* mutations compared with wild-type adrenocortical tissues [96]. Analysis of the RT-PCR primers employed by the authors suggests a selective upregulation of the *PDE2A1* transcript. These findings might conflict with expectations of reduced PKA activity, and therefore reduced steroidogenesis, upon elevated PDE expression. Notably, *PDE2A1* encodes the cytosolic isoform of phosphodiesterase 2A, which is distinct from its membrane-bound and mitochondrial counterparts. Given its subcellular localization, accumulation of PDE2A1 in the cytosol could distort cGMP/cAMP crosstalk within adrenal cortical cells. It is also possible that PDE2A1 upregulation is a compensatory mechanism to counteract signaling disruptions caused by aberrant *CTNNB1*. However, precise impacts of adrenal phosphodiesterases are currently obscure and the molecular mechanisms by which elevated cytosolic PDE2A1 expression may contribute to dysregulated cortisol production remain to be elucidated.

### 7.2. PDE8A/B

The PDE8 family comprises PDE8A and PDE8B, both of which are high-affinity, cAMP-specific phosphodiesterases and are found to be localized near mitochondria [97]. Upon genome sequencing of the *PDE8A/B* coding regions in patients affected with micronodular adrenocortical hyperplasia, a two-year-old girl was identified with a germline missense mutation in *PDE8B* (c.914A → C; p.His305Pro). This mutation was inherited from her father, who exhibited abnormal nocturnal cortisol levels [91]. Furthermore, in vitro experiments demonstrated that human embryonic kidney (HEK293) cells expressing the H305P mutant displayed approximately 3–3.5-fold higher intracellular cAMP levels compared to cells expressing wild-type PDE8B, indicating a marked loss of catalytic function [91].

A systematic genetic screening study of *PDE8B* variants in patients with diverse adrenocortical tumors, including PPNAD, AIMAH, secreting/non-secreting adenomas, and adrenocortical carcinomas, identified nine *PDE8B* variants (six of them novel) [98]. Among these, two variants (H391A and P660L) were predicted to be damaging mutations, while others were predicted to be benign or mildly deleterious according to in silico analyses. Functional validation through in vitro transfection experiments confirmed the pathogenicity of H391A and P660L, as both substitutions resulted in a significant decrease in PDE activity and a corresponding increase in intracellular cAMP levels compared to wild-type PDE8B in HEK293 cells [98]. PDE8B knockout (KO) mouse models exhibited elevated urinary corticosterone levels due to ACTH hypersensitivity, and PDE8B knockdown via shRNA led to an increase in *StAR* and *MC2R* transcript levels, indicating enhanced adrenal steroidogenic signaling [88]. Moreover, treatment with a PDE8-selective inhibitor (PF-04957325) caused an increase in basal PKA activity, whereas 3-isobutyl-1-methylxanthine (IBMX, a broad PDE inhibitor with poor PDE8 inhibition) did not elicit this effect, further supporting a role of PDE8B in modulating cAMP-PKA signaling in adrenal physiology [88].

Interestingly, in a study focused on analyzing the association between the transcriptomic profile and genetic background to identify the pathogenic mechanisms of Cushing’s syndrome, researchers identified a novel, non-recurring *AKAP13–PDE8A* fusion gene in a cortisol-producing adenoma sample [99]. The intrachromosomal rearrangement event spliced the 5′ end of *AKAP13* (containing exons 1–7) to the 3′ end of *PDE8A* (starting from exon 3). This fusion variant retained the conserved catalytic domain of PDE8A located at the C-terminus [99]. This rearrangement is also recurrently found in the SW480 human colon adenocarcinoma cell line and has been identified in primary colorectal cancer tissues [100]. AKAP13 (also known as AKAP-Lbc) is a scaffolding protein that anchors PKA and functions as a guanine nucleotide exchange factor (GEF) for Rho GTPases. AKAP13’s localization in adrenal tissue is not yet known, but in other tissues it has been shown to localize to the actin cytoskeleton via α-catulin and filamins A and B [101]. As discussed earlier, PDE8A proteins are typically localized to the mitochondria, where they regulate local cAMP concentrations. The *AKAP13–PDE8A* fusion could mislocalize PDE8A away from the mitochondria, leading to elevated cAMP levels in that region. This increase in cAMP would enhance PKA activity, resulting in excessive phosphorylation of StAR and ultimately promoting cortisol overproduction, a hallmark of Cushing’s syndrome. Empirically uncovering the mechanisms of the AKAP13-PDE8A fusion protein will provide exciting insights into adrenal cell physiology.

Finally, transcriptome analysis of 22 unilateral adrenocortical adenomas (including non-secreting, subclinical cortisol-producing, and cortisol-producing types) revealed a strong positive association between cortisol secretion and *PDE8B* expression. Subsequent Western blot analyses confirmed PDE8B protein overexpression in cortisol-producing adenomas compared to non-secreting adenomas. Interestingly, the PKA activity-to-cAMP ratio was also elevated in cortisol-secreting tumors [102]. This paradoxical relationship, wherein high PDE expression correlates with increased cortisol secretion, could again suggest compensatory PDE expression, or possibly that PDE8B plays non-canonical roles in steroidogenesis beyond simple cAMP degradation.

### 7.3. PDE11

Horvath et al. performed single-nucleotide polymorphism (SNP) genotyping of adrenocortical hyperplasia patients who lacked known driver mutations and identified variations in the chromosomal region 2q31–2q35 [103]. This region was found to encode *PDE11A*. The *PDE11* family comprises four isoforms (*PDE11A1–A4*), all of which encode proteins that exhibit dual specificity toward cAMP and cGMP [103]. Among these, PDE11A4 is the only isoform detected in the adrenal gland [104]. Western blot analyses of tumor tissues from affected patients with *PDE11A* mutations revealed a marked reduction in PDE11A4 protein levels compared to normal adrenal tissue. Functionally, these mutations led to elevated intracellular cAMP and cGMP levels, consistent with impaired phosphodiesterase activity [89]. Additionally, the authors observed a four-fold increase in phosphorylated CREB (pCREB) to total CREB ratios in tumor tissues, likely reflecting enhanced cAMP/PKA signaling due to reduced PDE11A-mediated degradation of cyclic nucleotides [89]. Collectively, these findings suggest that PDE11A mutations contribute to adrenocortical hyperplasia and Cushing’s syndrome through dysregulated cAMP/cGMP homeostasis and subsequent overactivation of PKA-dependent transcriptional pathways.

## 8. β-Catenin

Another major cause of cortisol-producing adenomas is mutations in *CTNNB1*, the gene for β-catenin [105]. β-Catenin is a key transcription factor in the canonical Wnt pathway and drives transcriptional programs associated with cell growth and proliferation. Its levels are normally tightly controlled through phosphorylation-dependent degradation. Under usual conditions, CK1 and GSK-3β phosphorylate its N-terminal region, marking it for ubiquitination and proteasomal destruction [106]. PKA phosphorylation at Ser-552 and Ser-675 stabilizes β-catenin, promotes its movement into the nucleus, and enhances transcription via TCF/LEF and coactivators like CBP [107,108]. PKA can also facilitate β-catenin degradation. Phosphorylation at Ser-45 primes β-catenin for further GSK-3β action, accelerating its breakdown and reducing transcriptional activity [108].

In adrenal development, PKA suppresses Wnt/β-catenin signaling to maintain proper zonation and prevent tumor formation [109]. In this process, sustained PKA activity limits β-catenin-driven proliferation, illustrating a tissue-specific antagonism between the two pathways. Activating mutations in *CTNNB1* that are found in Cushing’s syndrome patients most commonly affect exon 3 residues such as S37, T41, or S45. Substitutions at these amino acids prevent GSK-3β–mediated phosphorylation and block β-catenin degradation, resulting in constitutive β-catenin stabilization and nuclear accumulation. This results in persistent activation of Wnt target genes, which contribute to the formation of cortisol-producing adenomas [110,111]. *CTNNB1* alterations also drive aldosterone-producing adenomas and hyperplasia of the adrenal cortex and recent work has demonstrated that disrupting fibroblast growth factor signaling in these cells may offer therapeutic benefit [112,113].

## 9. PKA Regulatory Subunit Roles in Cushing’s Syndrome

One of the most important genes associated with hypercortisolism is *PRKAR1A*, the gene for PKA regulatory subunit RIα. Studies from the turn of this century discovered a strong relationship between dysfunctional RIα and Carney complex [114,115]. Carney complex patients often develop cortisol-producing adrenal tumors and suffer from Cushing’s syndrome [116]. It is expected that loss of kinase regulation by RIα results in overactive PKA-dependent transcription, which then drives both tumor formation and overproduction of steroidogenesis machinery such as StAR. Indeed, adrenal-specific loss of RIα in a mouse led to elevated PKA activity, hyperphosphorylated CREB, suppressed apoptosis, and overproduction of glucocorticoids [117,118]. Approximately 80% of Carney complex patients carry mutations in *PRKAR1A* [119].

Variants of the other type I PKA regulatory subunit, RIβ, have also been found in cortisol-producing adenomas and bilateral adrenocortical hyperplasia [120]. Point mutations (I40V, A67V, and A300T) were identified in 3 out of 74 patients diagnosed with isolated micronodular adrenocortical disease and Cushing’s syndrome. Copy number gains of *PRKAR1B* were also identified in adrenal adenomas (3 out of 21 patients) including one driven by the most prevalent catalytic subunit mutation L205R. The distinction of these *PRKAR1B* variants versus *PRKAR1A* lesions is that the changes in RIβ present as compensatory alterations that decrease PKA activity. The two conserved point mutations, A67V and A300T, both increase affinity for the catalytic subunit and lower its basal activity [120].

Additional work has looked at the different contributions of PKA regulatory subunits in adrenal tumors. The first study to systematically examine relative R subunit expression levels in non-Carney complex cases of adrenal Cushing’s syndrome found a selective downregulation of RIIβ in all samples, resulting in an approximate 80% decrease compared to normal adrenal tissue [121]. This was in stark contrast to both RIα and RIIα, which showed no significant differences. Experiments using R subunit-selective cAMP analogs demonstrated functional differences between the two types of PKA. RIα activation exhibited a stimulatory effect on Y1 adrenal cell proliferation while activation of RIIβ decreased cell numbers. This consequence was tested genetically as well, with depletion of RIIβ by siRNA causing an increase in Y1 cell proliferation [121]. However, knockdown of the RII isoform was accompanied by strong increases in RIα levels, occluding clear mechanistic conclusions. Downregulation in RIIβ was later tied to the presence of mutant *PRKACA* and shown to occur post-transcriptionally [122]. This study demonstrated that phosphorylation of serine 114 in the kinase inhibitory sequence labels RIIβ for degradation by caspase 16 [123]. Although specificity for RIIβ downregulation in adrenal tumors had been previously established, this mechanistic study was surprising because type II regulatory subunits are often thought of indiscriminately. These findings call for future studies into how the alpha and beta isoforms of RII differ in adrenal functions.

## 10. PKA Catalytic Subunit Mutations in Cushing’s Syndrome

Since 2014, attention regarding cAMP signaling in Cushing’s syndrome has largely focused on the PKA catalytic subunit (PKAc). Four articles published that year independently reported a high association of adrenal cortisol-producing adenomas with a somatic mutation in *PRKACA,* the gene that encodes the alpha isoform of PKAc [81,124,125,126]. This missense mutation, L205R, was identified in ~45% of cumulative patients and was associated with overt symptoms of Cushing’s syndrome and smaller tumor size. Since then, eight less prevalent variants of *PRKACA* have been associated with adrenal Cushing’s syndrome. These include the mutations E31V, W196G and W196R, insertions 198_199insW and 199_200insV, an S212R mutation with IILR insertion, a deletion of 243–247 with an E248Q mutation, and a deletion of 243–249 with Q insertion [105,127,128,129]. Additionally, one PKAcβ (encoded by *PRKACB*) mutation, S53L, has been identified as a driver of Cushing’s syndrome [130].

**Figure 5:**
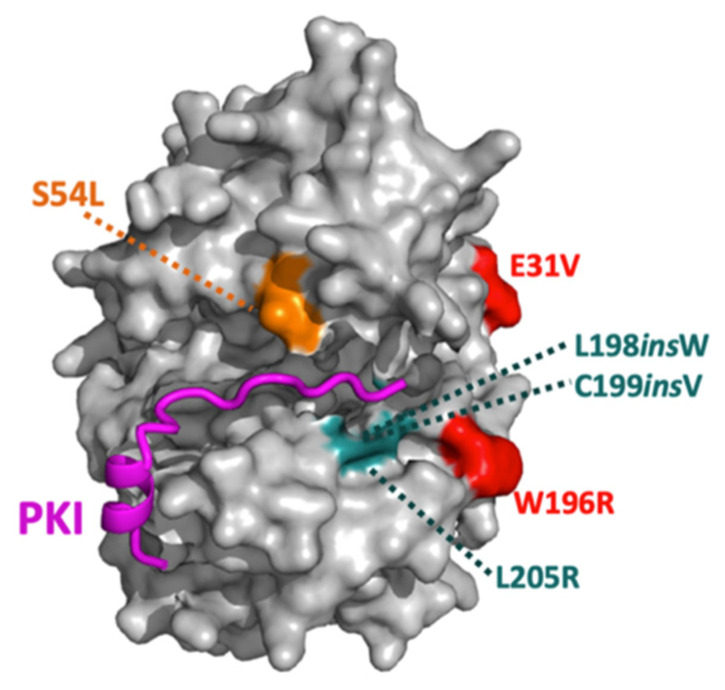
Modeling PKAc-PKI binding disruptions in Cushing’s syndrome. Structural depiction of Cushing’s PKAc mutations in relation to the PKI binding interface. From [132].

Comparative studies have revealed surprising differences among Cushing’s PKAc mutants in terms of kinase structure and function and likely mechanisms of pathogenesis (Table 1) [131]. For instance, a combination of biochemical and cell-based assays determined that five of these mutant kinases fall into two different categories [132]. This study showed that the E31V and W196R Cushing’s PKAc variants bind the endogenous PKA inhibitor PKI and are therefore excluded from the nucleus. The other tested variants, 198_199insW, 199_200insV, and L205R, all fail to bind PKI and therefore populate the nuclear compartment. Kinases in the former group behave similarly to the wild-type kinase in this regard and appear to have higher levels of catalytic activity. This study also used modeling of the kinase bound to a PKI peptide to speculate that the two categories of mutants diverge due to the physical placement of the mutations in reference to the bound inhibitor peptide (Figure 5) [132]. For instance, because E31V and W196R do not occur within the main PKI binding interface, they might not disrupt binding and subsequent nuclear export. Under this model, one would expect the 243–247del/E248Q variant to segregate with non-PKI binders and the S212R/insIILR variant to possibly retain association with PKI. The PKAcβ-S53L Cushing’s mutant could disrupt interactions between the catalytic subunit and PKI, as the mutant leucine likely points down toward the pseudosubstrate. The PKAcβ variant also disrupts a conserved autophosphorylated serine [133]. Loss of this charged post-translational modification could certainly impact substrate and PKI interactions. A subsequent report included tests of a novel variant, PKAc-W196G, which displayed behaviors similar to its counterpart, PKAc-W196R, including nuclear exclusion and retained binding to PKI [134].

Nuclear Magnetic Resonance studies delved into the more detailed structural impacts of Cushing’s mutations [135]. This group found that the L205R substitution not only disturbs the substrate-binding interface but also disrupts the internal allostery of the kinase. R205 falls within an “allosteric node” that connects the N and C lobes of the kinase. Perturbations caused by this mutation reduce both the cooperativity of the two lobes and the enzyme’s affinity for ATP. Interestingly, the authors use modeling to show that these highly coupled allosteric nodes contain all of the identified PKAcα Cushing’s mutations, and therefore may create a theoretical model that explains the similar phenotypes observed among disparate mutations [135]. An additional study by this group examines the E31V and W196R mutants in a similar way and concludes that both amino acid substitutions mimic the effects of L205R on allosteric cooperativity [136]. While it is attractive to hypothesize that disruptions to PKAc nucleotide binding could help explain the observed catalytic activity deficiencies of certain Cushing’s mutants, this might not be the case as the W196R variant closely resembles the wild-type kinase in terms of structural stability and catalytic activity [132,133,136].

Synthesis of the differential R subunit binding displayed by PKAc variants also provides insight into the possible general mechanisms underlying Cushing’s pathology [131]. Most of the identified Cushing’s variants tested for R subunit binding have exhibited deficient binding to RI, ranging from completely abolished to intermediate association (Table 1). Conversely, two of the variants have shown no loss of association with RII. These data suggest that healthy regulation of cortisol production may require full affinity for RI and that deficient interaction with RI can override normal binding to RII. Investigations into the spatiotemporal aspects of these findings are needed to increase our understanding of dynamic PKA regulation, both in the adrenal gland and in general.

The majority of studies regarding PKAc regulation by R subunits and the kinase’s downstream pathogenic actions have focused on the most prevalent L205R mutant. Several groups have now shown that PKAc-L205R fails to bind PKA regulatory subunits [81,126,133,137,138]. This results in an enzyme that is unresponsive to cAMP and constitutively active [124]. Interestingly, the point mutation also disrupts the catalytic efficiency of the enzyme, rendering PKAc-L205R 6–20× less active than its wild-type counterpart [133,138,139]. Lack of R binding also causes the mutant kinase to be dissociated from its normal subcellular targets [133]. In this study, the use of biochemical assays in conjunction with live-cell proximity biotinylation and photoactivation mobility measurements established that both L205R and W196R variants lack association with adrenal AKAPs. Kinase mislocalization was apparent in stained patient tissue as well. Whereas PKAc localized mainly to the cell membrane in non-tumor adrenal tissue, it was diffuse throughout the cytoplasm in tumor sections [133].

Cell component analysis of proximity proteomic results demonstrated that PKAc-L205R is more associated with both nuclear and mitochondrial machinery compared to the wild-type kinase [133]. As discussed above, these subcellular components are involved in transcriptional regulation and phosphorylation of StAR, respectively [68]. Perhaps enhanced association of PKAc-L205R with these subcellular regions overcomes the mutant’s poor catalytic efficiency by focusing its weak efforts on key points in cortisol production. The W196R variant also demonstrated increased mitochondrial associations. However, this Cushing’s variant did not demonstrate nuclear enrichment despite strongly increased StAR levels in W196R cells. Since it has compromised interactions with regulatory subunits but retains binding to the nuclear exporter PKI, it is possible that PKAc-W196R enters the nucleus more than wild-type PKAc, but is quickly shuttled out [132,133,140,141]. Impacts on *StAR* transcription could likely still occur during this brief exposure as the W196R variant displays near-wild-type levels of catalytic activity [133]. Further empirical studies of all Cushing’s mutants are needed to fully understand the functional consequences of subtle differences in mislocalization.

It is presumed that the somatic PKAc mutations are heterozygous. Thus, one intact allele may avoid most possible loss-of-function impacts of PKAc-L205R mislocalization. Gain-of-function impacts likely include increased or constant steroidogenic signaling via enhanced phosphorylation of on-target substrates. Gain-of-function alterations could also involve off-target phosphorylation of amenable aberrant substrates. PKA is a promiscuous kinase that is able to phosphorylate most proximal basophilic substrates (containing R or K at positions upstream of the phosphorylated serine or threonine) [23,142]. Interestingly, substitution of an arginine at L205 mimics structural components of proline-directed kinases and may bias the mutant enzyme toward atypical substrates [143]. Thus, abnormal phosphorylation targets in L205R tumors could include both basophilic substrates normally modified by other basophilic kinases and more divergent substrates normally targeted by different classes of kinases. Indeed, PKAc-L205R’s substrate preference has been shown to be skewed toward substrates containing acidic sidechains at positions immediately downstream of the phosphorylated residue [135,144,145]. While empirical evidence for substrate rewiring by an L205R substitution has been shown by multiple groups, the impacts of these potential changes regarding Cushing’s pathology have yet to be firmly established [133,144,145]. Regardless, PKAc-L205R substrate studies have produced two promising candidates for PKAc-L205R effectors: histone H1.4 modification and upregulation of YAP/TAZ levels [133,145].

Histone H1.4 is phosphorylated on serine 36 during cell cycle progression to promote dissociation from chromatin [146]. This site was identified as hyperphosphorylated in an unbiased screen for PKA substrates associated with PKAc-L205R [145]. In these experiments, mutant or wild-type PKAc was expressed in HEK293A cells and then incubated with the adenylyl cyclase activator forskolin for 30 min. Lysates were then subjected to immunoprecipitation with an antibody to the phosphorylated PKA substrate recognition motif (RRXS/T) and processed for proteomic analysis. Mass spectrometry identified H1.4 as over 2× phosphorylated in L205R conditions compared to wild-type. Subsequent evaluation of patient adrenal tumors corroborated this evidence by finding elevated phospho-S36 levels in three L205R patients compared to four samples from patients without the L205R mutation [145]. Experiments to identify the kinase for H1.4 S36 screened ten candidate basophilic and mitotic kinases by overexpression in HEK293T cells and found PKA as the only of these to phosphorylate histone H1.4 S3 [146]. While these experiments did not recapitulate normal protein levels and native signaling environments, they established that PKA is able to modify this histone. Irrespective of whether the event is part of normal cell proliferation, the combined evidence from Bathon et al. and Chu et al. suggests that PKAc-L205R-mediated phosphorylation of H1.4 could be driving tumor formation in adrenal Cushing’s syndrome patients. Future work will need to isolate upregulation of H1.4 S36 phosphorylation in mouse models and adrenal cells to ascertain its impact on glucocorticoid production and tumor formation.

YAP/TAZ signaling also regulates tissue growth and may be involved in Cushing’s pathology. YAP and TAZ are transcriptional co-regulators that interact with the TEA domain family of proteins (TEAD) to induce expression of genes that promote cell proliferation and counteract apoptosis [147]. YAP and TAZ are highly active during tissue growth during development and have been implicated in numerous cancers [148]. In differentiated cells, activity of YAP and TAZ is inhibited by the Hippo pathway. The scaffold protein disks large homolog 5 (DLG5) inhibits Hippo pathway repression of YAP/TAZ, therein disinhibiting proliferation [149]. In a proximity phosphoproteomic screen performed in H295R human adrenal cells, DLG5 was identified as hyperphosphorylated in the PKAc-L205R condition [133]. Subsequent experiments demonstrated increased YAP and TAZ levels in PKAc-L205R adrenal cells compared to control cells expressing the wild-type kinase. Further analysis of clinical samples from Cushing’s patients also revealed increased YAP and TAZ in tumors versus non-tumor adrenal tissue [133]. While increased levels of these transcriptional co-activators could explain tumor growth, a more direct impact on steroid synthesis is also possible. Future work needs to assess the mechanistic details of PKAc-L205R enhancement of YAP/TAZ levels and the clinical relevance of YAP/TAZ overexpression in Cushing’s syndrome.

Interestingly, identical proximity phosphoproteomic experiments with the W196R Cushing’s variant of PKAc showed no increases in YAP or TAZ levels. Moreover, confirmation of this finding in an adrenal-specific W196R mouse model showed a significant decrease in YAP and TAZ in the adrenal cortex of mutant mice, concurrent with an increase in ERK1/2 phosphorylation [133]. These observations present the possibility that similar mutant kinases driving the same disease promote tumor growth via two different mitogenic pathways. This also raises the question of why the two signaling aberrancies do not coincide. While a report of ERK-mediated downregulation of YAP exists, studies are needed to better understand both the mechanism of phospho-ERK upregulation by PKAc-W196R and how this increased activation might counteract YAP/TAZ signaling [150].

**Table 1:** Functional consequences of PKA catalytic subunit mutations found in Cushing’s syndrome. Inhibitory interactions assessed include RI, RII, and PKI, and resemble those of the WT kinase unless otherwise stated. Studies referenced: [123,125,130,132,133,134,136,145,151].

Mutations	Inhibitory Interactions	Signaling Disruptions
E31V	RI inhibition weakly to not disrupted	
W196R	RI inhibition moderately disrupted; RII inhibition strongly disrupted	Upregulated pERK and decreased YAP/TAZ levels (cells and knock-in mice); increased RIα and decreased RIIβ levels (cells and knock-in mice); increased StAR levels (cells)
W196G	RI inhibition weakly disrupted; RII inhibition strongly disrupted	Increased RIα levels (patient tissue)
L198insW	RI, RII, and PKI inhibitions strongly disrupted	
C199insV	RI and PKI inhibitions strongly disrupted; RII inhibition moderately disrupted	
L205R	RI, RII, and PKI inhibitions strongly disrupted	Increased YAP/TAZ levels, increased histone H1.4 phosphorylation, increased StAR levels (cells and patient tissue); decreased RII levels (cells and patient tissue)
S212R + insIILR	RI and RII inhibitions strongly disrupted; no data on PKI	
del243–247 + E248Q	RI and RII inhibitions strongly disrupted; no data on PKI	
S53L (PKAcβ)	RI and PKI inhibitions moderately disrupted	

## 11. Other cAMP Effectors

In addition to PKA, there are other protein families whose actions are regulated directly by cAMP. The most studied of these in the adrenal gland are exchange proteins directly activated by cAMP (EPACs). The EPAC family consists of two major isoforms, EPAC1 (encoded by *RAPGEF3*) and EPAC2 (encoded by *RAPGEF4*), and include multiple transcripts expressed in different tissues [152]. Among these, EPAC2B is the only isoform described in the adrenal gland [153]. To elucidate the contribution of EPAC2 to steroidogenesis, Lewis and colleagues performed a series of experiments using cAMP analogs that have been shown to selectively activate either PKA or EPAC [154]. Their findings provided evidence that PKA-selective cAMP analogs increased the expression of StAR, CYP11A1, and CYP17 in H295R cells, whereas EPAC-selective analogs had no effect on steroidogenesis machinery. Furthermore, the authors demonstrated that PKA, but not EPAC2B, induced the expression of the NGFI-B nuclear receptor, which is involved in the transcriptional regulation of steroid hydroxylase genes [154]. It is important to note here that while EPAC-selective analogs offer the benefit of a lower effective nucleotide concentration for in vitro experiments, EPAC activation generally requires much higher cAMP concentrations than does PKA activation and this should be considered when working in endogenous systems [71]. Interestingly, a more recent study in mice reported upregulation of adrenal EPAC2 during the ACTH-independent production of glucocorticoids in response to acute infection, suggesting an alternative cAMP-dependent path to steroidogenesis in certain environmental contexts [155].

Cyclic nucleotide-gated (CNG) channels can also respond directly to local increases in cAMP by altering the conductance of ions. While expression atlas data suggest detection of both transcript and protein for hyperpolarization-activated cyclic nucleotide—gated channel 3 (HCN3) in the adrenal cortex, we found no studies of CNG channels in adrenal steroidogenesis [156,157]. Similarly, Popeye domain-containing (Popdc) proteins are membrane proteins that have high affinity for cAMP and have been found to control important structural and functional properties in both skeletal and cardiac muscle [158]. To our knowledge, no functions for Popdc proteins have been reported in adrenal cells. Future studies of cAMP functions in adrenal physiology may benefit from considering possible roles of alternative effectors.

## 12. Closing Remarks

Over half a century of work has revealed much about how ACTH triggers cortisol production from adrenal zona fasciculata cells. While cAMP and PKA were both identified as crucial mediators of steroidogenesis decades ago, elucidation of mechanistic details in this pathway has proved elusive. However, a resurgence of efforts from the last ten to fifteen years has generated novel findings and shows promise of continued efforts to understand this important physiological process. Future work should utilize advanced, physiologically relevant techniques to incorporate recent revelations regarding PKA signaling into our current understandings of adrenal function in health and disease. Importantly, much of the work performed in order to understand cAMP-PKA pathway dysregulation in adrenal disorders also sheds light on how this kinase functions in healthy adrenal glands. Further investigations into both normal and disease conditions will reveal deeper insights into the actions of PKA in cortisol production.

## Data Availability

No new data were created or analyzed in this study.
